# Telocytes in ileum of the Chinese giant salamander: ultrastructural evidence

**DOI:** 10.1111/jcmm.12741

**Published:** 2016-01-25

**Authors:** Hui Zhang, Shengwei Zhong, Tingting Ge, Shasha Peng, Pengcheng Yu, Zuohong Zhou, Xiaoquan Guo

**Affiliations:** ^1^ College of Animal Science and TechnologyJiangxi Agricultural UniversityNanchangChina

**Keywords:** telocytes, telopodes, ileum, giant salamander, ultrastructure

## Abstract

Telocytes (TCs) and their telopodes (Tps) have been found in various organs of many mammals, including in lower animals. However, knowledge of TCs in lower animals is still very limited. This study identified TCs and their Tps in the ileum of the Chinese giant salamander, *Andrias davidianus* (Amphibia: Caudata), by transmission electron microscopy. The TCs/Tps were found near epithelial cells, glandular cells and unmyelinated nerve fibres. Moreover, exosomes were also found to be present in between TCs/Tps and these cells.

Many investigations have documented that TCs and their Tps are resident in various organs of many mammals, including human, pig, gerbil, mouse, rat and degus 1, 2, 3, 4, 5. Studies on TCs and Tps have also been reported in lower animals, such as newts, zebrafish and turtles 6, 7. However, knowledge about TCs in lower animals is still very limited. In this study, we identified the TCs/Tps in the Chinese giant salamander, *Andrias davidianus* (Amphibia: Caudata), using transmission electron microscopy (TEM) to improve our understanding of amphibian tissue regeneration 8.

After euthanizing on the ice, four farmed (two males and two females), 2.5‐year‐old Chinese giant salamanders (weight: 0.99–1.12 kg) were killed, and the ileums were excised. Small pieces of ileum were fixed in 2.5% glutaraldehyde/PBS. The specimens were sectioned with a LKB‐V ultramicrotome (Bromma, Stockholm, Sweden). The ultrathin sections were observed and photographed using a JEM‐1200EX TEM (JEOL, Tokyo, Japan).

In the TEM images, TCs and their Tps segments were located in the lamina propria of the ileums from the Chinese giant salamander (Figs 1, 2, 3, 4). TCs had polygonal (Fig. 1) or spindle‐shaped (Figs 2 and 3) cell bodies containing a large nucleus and scanty cytoplasm. TCs usually had 2–3 Tps. TCs/Tps were located adjacent to epithelial cells and glandular cells (Figs 1 and 2). Moreover, the exosomes were frequently present between TCs/Tps and these cells (Figs 2 and 3). One TC/Tp and another TC/Tp were connected by close contact (Figs 1 and 3). TCs were also observed in the vicinity of unmyelinated nerve fibres (Fig. 4). The cytoplasmic processes of Schwann cell surrounded the axons, which contained synaptic vesicles, mitochondria and microtubules (Fig. 5).

**Figure 1:**
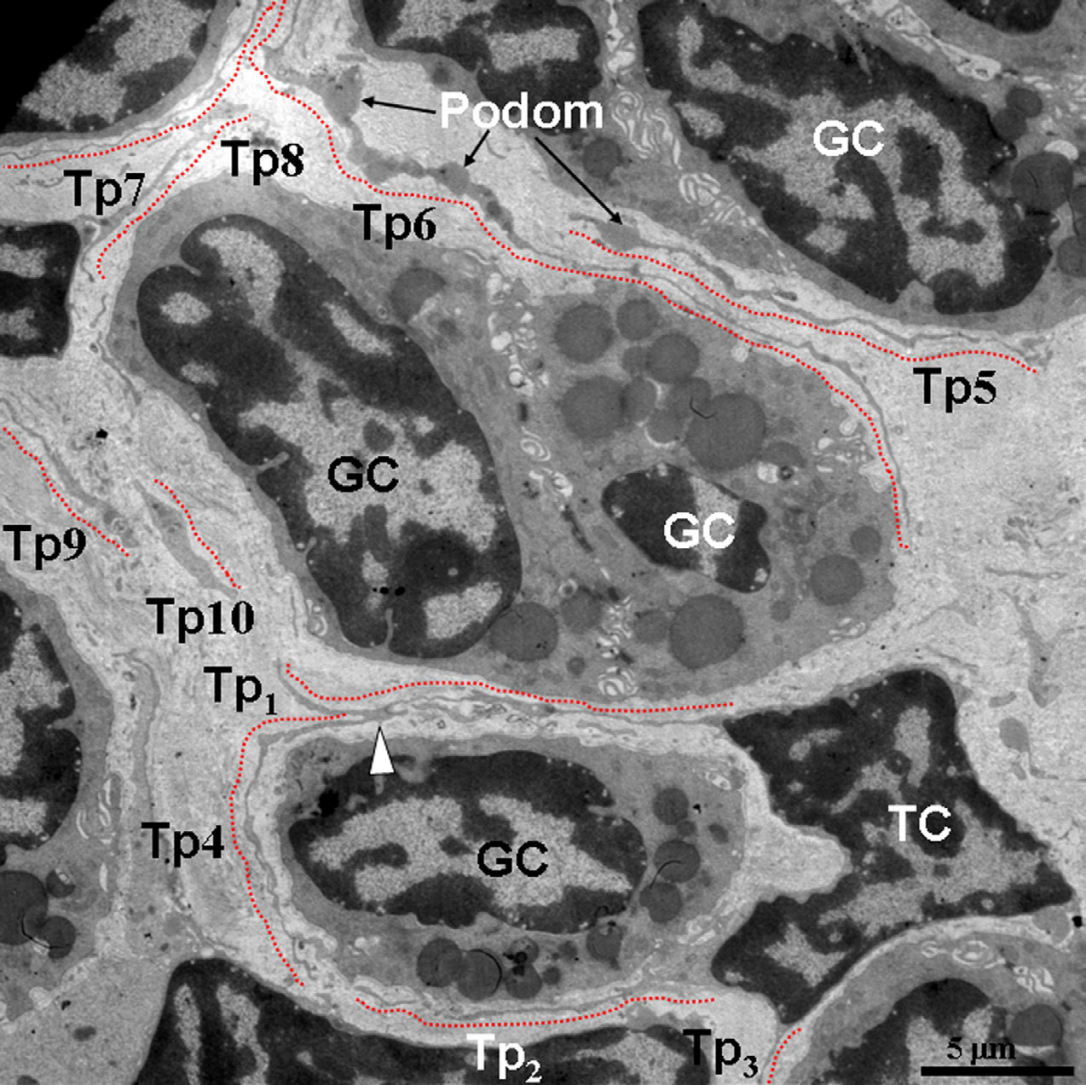
Telocytes (TCs) and their telopodes (Tps) were present between glandular cells (GC). A TC with three Tps (Tp_1_, Tp_2_ and Tp_3_) and Tps indicated in red dashed lines were observed. Close contacts were observed between two Tps (white arrowhead). A GC was surrounded by Tps of the TC. The Tps with long, tortuous prolongations and uneven calibre (moniliform), podoms and podomers were present. The cytoplasm of the GC contained electron‐dense, homogeneous and rounded gland granules.

**Figure 2:**
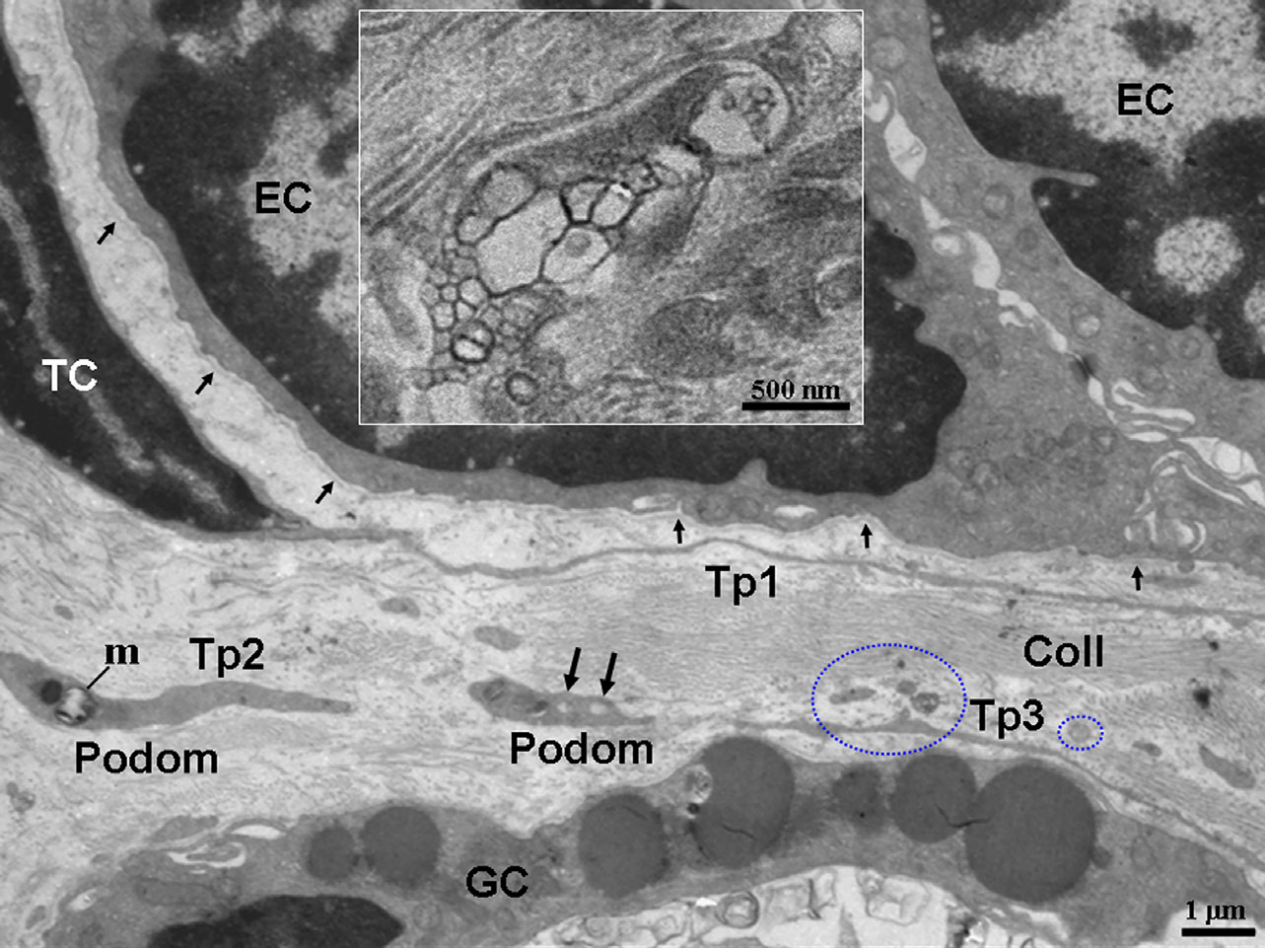
TCs/Tps were located between glandular cells (GC) and epithelial cells (EC). A TC with long and thin Tp1; the podom of the Tps contained mitochondria (m) and caveolaes (black long arrows). Exosomes (blue circles) were also observed. The EC with a thin and long basal lamina (black short arrows) are shown. The inset shows magnified exosomes. Coll, collagen fibres. TC: telocyte; Tp: telopode.

**Figure 3:**
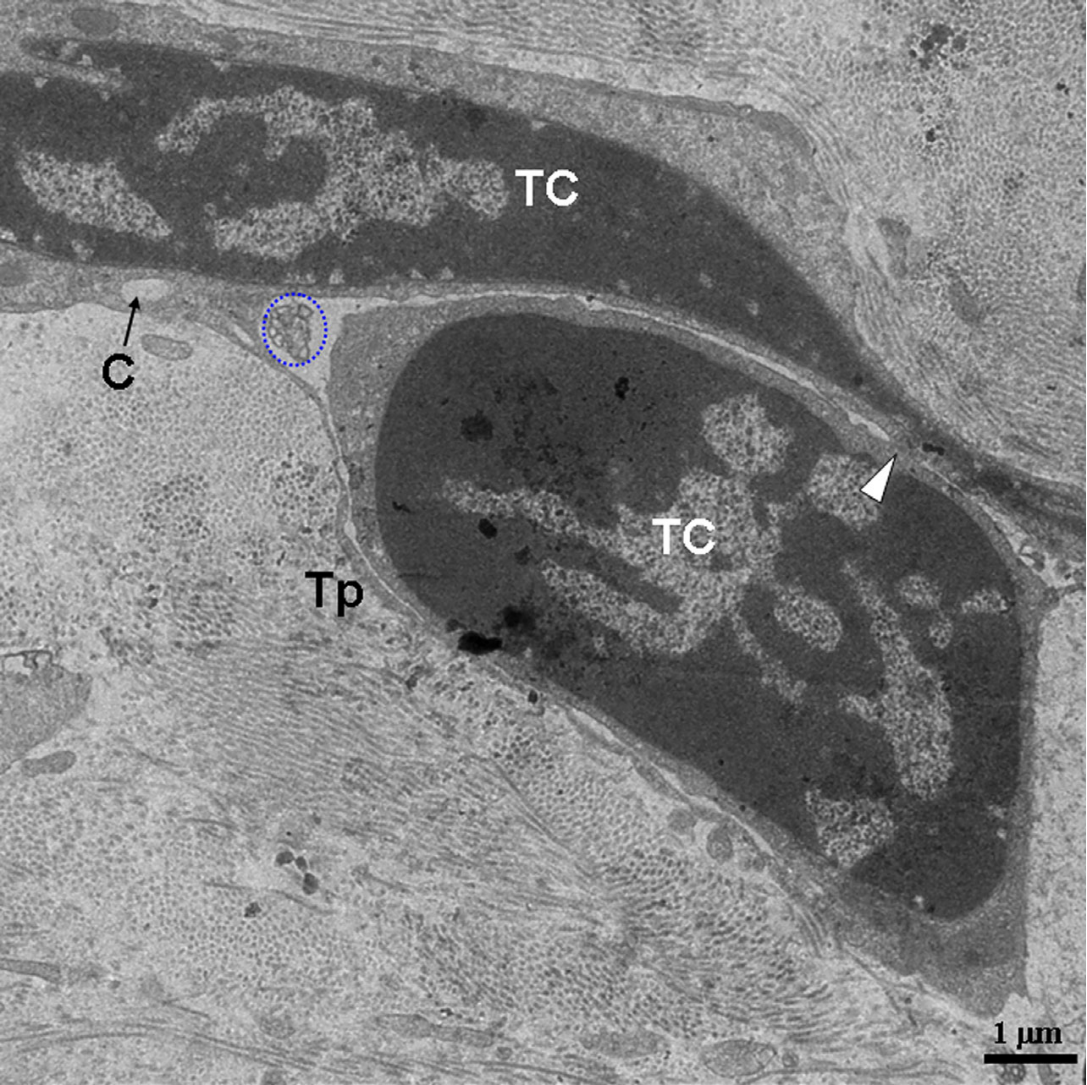
Two TCs are in close proximity. A TC with a thin, long Tp surrounds another TC. The white arrowhead indicates close contact; the blue circle indicates exosomes. TC: telocyte; Tp: telopode; C: caveolae.

**Figure 4:**
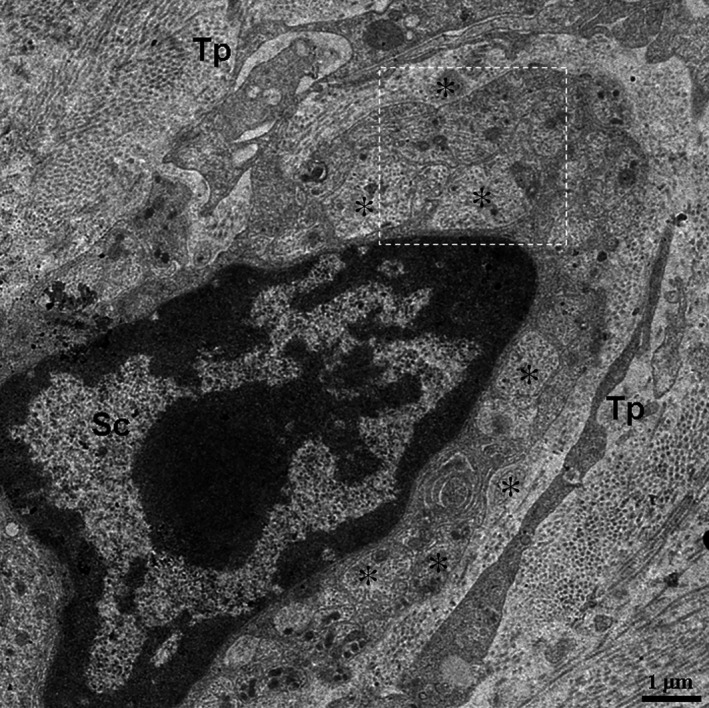
The location of Tps in close proximity to an unmyelinated nerve fibre. The asterisk indicates axon. TC: telocyte; Tp: telopode; Sc: Schwann cell.

**Figure 5:**
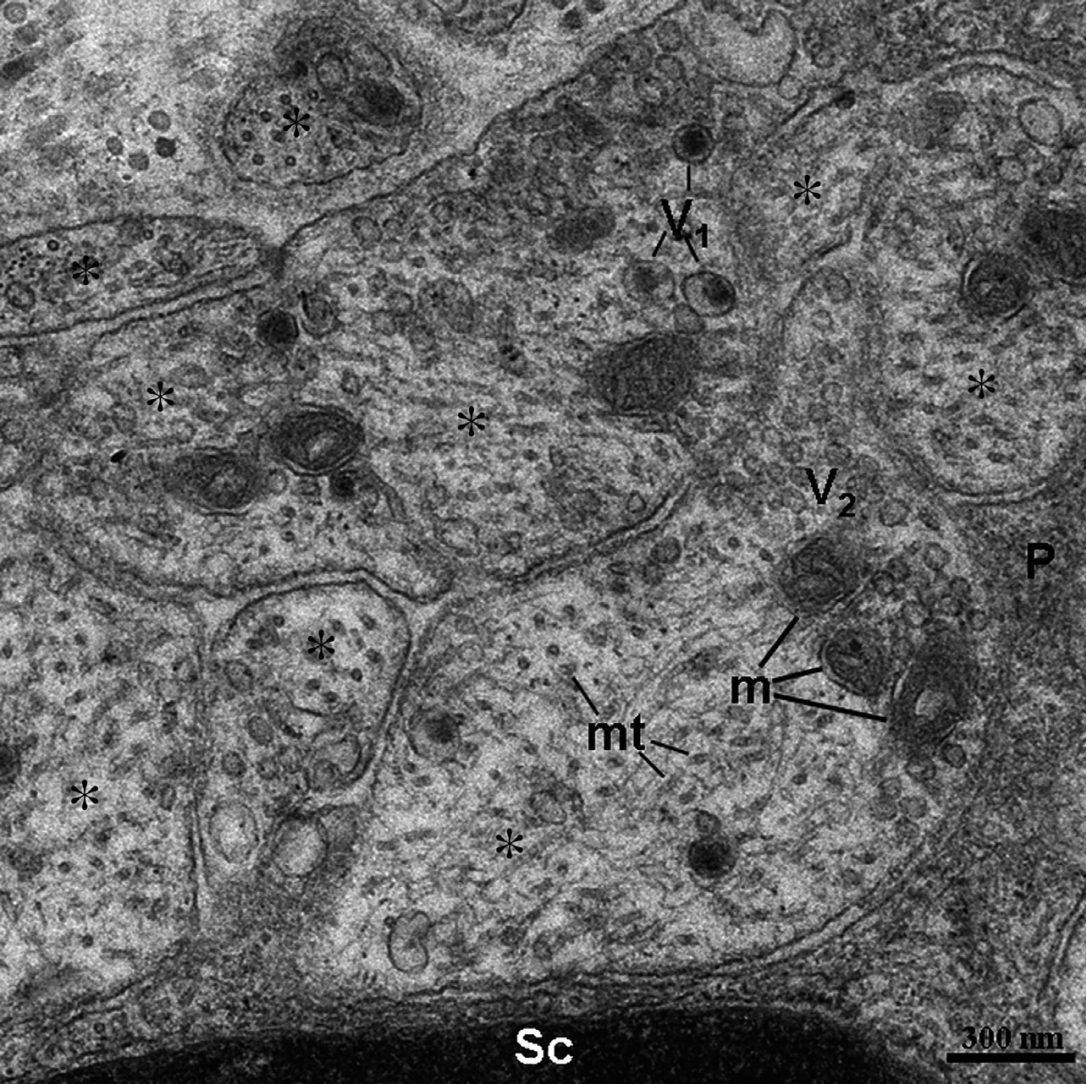
High magnification TEM image of the dashed line boxed areas shown in Figure 4 with details of axons. The axons contained two types of vesicles. The V_1_ type of vesicle possessed an electron‐dense core. The V_2_ type of vesicle shows an electron‐lucent vesicular‐shaped structure. The asterisk indicates axon. Sc: Schwann cell; m: mitochondria; mt: microtubules; P: cytoplasmic process of Schwann cell.

In the previous studies, TCs were identified in gastrointestinal system of mammals, for example human, mice and rats 9, 10, 11, 12, 13, 14. However, the roles of TCs in the gastrointestinal system are still imperfectly elucidated. Some studies indicate TCs are potentially involved in liver growth and regeneration 13, 14. TCs could be also involved in intercellular signalling, immune response and control of tissue homeostasis in intestinal tract 9, 10. In this study, TCs/Tps were observed in the vicinity of epithelial cells, glandular cells and unmyelinated nerve fibres of ileum. These results suggest that the cells/nerves might have interactive biological functions. The previous studies demonstrate that TCs cooperate with stem cells to induce tissue repair and regeneration in the gastrointestinal tract 10. Therefore, TCs might be involved in renewal of the gut epithelium in amphibians. TCs coexisted with glandular cells and serve coordinated physiological functions. It is suggested that TCs regulate the secretion of glandular cells 2. TCs might also play a role in glandular cells regeneration of ileum as TCs in another digestive gland—liver 13, 14. Moreover, TCs might play important roles in the maintenance of glandular homeostasis 6, 15. Likewise, TCs might contribute to control some physiological responses in the gut, hence their close proximity to nerve fibres 2. Exosomes were also found near TCs/Tps. These results suggest that the exosomes released from TCs/Tps could play a key role in regulating neighbouring cells 10.

## Conflicts of interest

The authors declare that there are no conflicts of interest.
